# From policy to practice: why the WHO’s Africa rehabilitation strategy 2025–2035 risks failure without educational reform

**DOI:** 10.3389/fmed.2025.1716773

**Published:** 2026-01-06

**Authors:** Ibrahim Npochinto Moumeni

**Affiliations:** 1 Department of Physiotherapy and Physical Medicine, Faculty of Medicine and Pharmaceutical Sciences, University of Dschang, Dschang, Cameroon; 2 Department of Physical Medicine and Osteopathy, Bafoussam Regional Hospital, Bafoussam, Cameroon; 3 Faculty of Medicine, Sorbonne University, Paris, France; 4 Faculty of Medicine, University of Parakou, Parakou, Benin; 5 Franco-African Centre for Applied Rehabilitation and Health Sciences (CFARASS), Foumbot, Foumbot, Cameroon; 6 Institut des Neurosciences Appliquées et de Rééducation Fonctionnelle: Institute of Applied Neurosciences and Functional Rehabilitation (INARF), Yaoundé, Cameroon; 7 Francophone African Society for Neurorehabilitation (SAFnER), Parakou, Benin

**Keywords:** health policy implementation, medical education reform, rehabilitation services, health systems strengthening, workforce development, sub-Saharan Africa, curriculum integration, WHO strategy

## Abstract

**Background:**

In July 2025, the WHO African Regional Committee adopted an ambitious strategy to address the 63% rehabilitation access gap through a comprehensive five-pillar framework. However, systematic educational exclusion of rehabilitation in African medical curricula may undermine implementation across all strategic pillars.

**Methods:**

Analysis of the WHO AFRO 2025–2035 strategy implementation framework, complemented by systematic curriculum assessment across Central African medical schools and ethnographic observations from Cameroon documenting current access barriers and workforce knowledge gaps.

**Results:**

Medical schools across Central Africa systematically exclude rehabilitation from curricula, with the University of Dschang representing a rare exception (4 h annually). This educational vacuum generates cascading failures: policymakers cannot prioritize services they don’t understand (Pillar 1: Governance), physicians cannot refer to specialists they’ve never encountered (Pillar 2: Workforce), evidence-based interventions are dismissed in favor of pharmaceuticals (Pillar 3: Service Delivery), rehabilitation needs remain invisible in data systems (Pillar 4: Information), and financing mechanisms struggle to support undervalued services (Pillar 5: Resources). Field evidence reveals patients traveling over 1,000 km for rare rehabilitation expertise, reflecting profound system-level educational failures.

**Conclusion:**

Without urgent educational reform, the WHO strategy risks replicating historical implementation failures despite comprehensive policy architecture. Educational exclusion represents not a peripheral concern but a foundational threat to strategy success. Medical curriculum integration offers a cost-effective, scalable intervention that amplifies all five strategic pillars. African health leaders must prioritize educational reform as essential infrastructure, not optional enhancement.

## Introduction

The WHO African Regional Committee’s July 2025 resolution represents either a watershed moment for rehabilitation in Africa—or another well-intentioned policy framework destined for implementation failure (1, 2). The Regional Strategy to Strengthen Rehabilitation in Health Systems 2025–2035 acknowledges that over 63% of Africans lack access to essential rehabilitation services, a crisis affecting an estimated 450 million people across the continent (2, 3).

### The arithmetic of neglect is stark

With rehabilitation workforce density at 0.04 per 10,000 in Sub-Saharan Africa versus 0.7 globally, achieving WHO targets by 2035 requires training approximately 280,000 new rehabilitation professionals across the continent (4–6). Yet systematic curriculum assessment reveals that 87% of Central African medical schools allocate zero hours to rehabilitation training, while the remaining 13% offer <5 h annually [present study data]. This educational exclusion predicts implementation failure with mathematical certainty: you cannot staff what students have never encountered, cannot refer appropriately to specialists physicians don’t understand, and cannot integrate services that policymakers cannot conceptualize.

This unprecedented policy framework organizes interventions around five strategic pillars: (1) governance and leadership, (2) workforce development, (3) service delivery expansion, (4) information systems, and (5) resource mobilization (2). The architecture is comprehensive, evidence-based, and technically sound. Yet a critical question remains unaddressed: Why should this strategy succeed where previous rehabilitation initiatives have failed?

The answer—or rather, the warning—emerges from systematic examination of medical education across Africa. The educational systems producing Africa’s healthcare workforce remain largely disconnected from rehabilitation principles, potentially perpetuating the very professional knowledge gaps that contributed to the current access crisis (4, 6).

## The five-pillar strategy and its Achilles’ heel

### The comprehensive framework

The WHO AFRO strategy’s five pillars address rehabilitation systematically (2):

*Pillar 1—Governance and Leadership:* Strengthening policy frameworks, establishing national rehabilitation coordination mechanisms, integrating rehabilitation into essential health benefit packages.

*Pillar 2—Workforce Development:* Building capacity for multidisciplinary rehabilitation teams through pre-service and in-service training, addressing the critical shortage of rehabilitation professionals.

*Pillar 3—Service Delivery Expansion:* Expanding rehabilitation services at primary, secondary, and tertiary levels with emphasis on community-based approaches and functional integration.

*Pillar 4—Information Systems:* Developing data collection mechanisms to monitor rehabilitation needs, service coverage, quality indicators, and population outcomes.

*Pillar 5—Resource Mobilization:* Identifying sustainable financing mechanisms, optimizing resource allocation, and demonstrating cost-effectiveness to secure continued investment.

### The unacknowledged dependency

Each pillar implicitly assumes a workforce capable of understanding, valuing, and implementing rehabilitation interventions. This assumption may be fundamentally flawed.

Systematic assessment of medical education across Central Africa reveals that major medical schools—from the Universities of Yaoundé and Douala to Garoua—largely exclude rehabilitation from their core curricula (4, 7). The University of Dschang, ranked first in the CEMAC zone, represents a notable exception, offering minimal 4-h annual rehabilitation exposure (7). Even this limited exposure has demonstrated measurable impact on student career interests, suggesting the magnitude of opportunity lost through systematic educational exclusion.

## Why educational exclusion threatens all five pillars

### Pillar 1 failure mechanism: governance without understanding

Policymakers and health system leaders trained without rehabilitation exposure struggle to prioritize it in health planning (8, 9). Studies from Morocco document that even senior training doctors demonstrate significant knowledge gaps regarding Physical Medicine and Rehabilitation as a medical specialty (10). When decision-makers cannot comprehend rehabilitation’s role in managing Africa’s dual disease burden—persistent communicable diseases alongside rising non-communicable conditions—they default to pharmaceutical and surgical interventions despite rehabilitation’s superior cost-effectiveness for many conditions (3, 6).

#### The institutional isomorphism trap

Health systems tend to replicate familiar organizational structures (8). Without educational exposure to rehabilitation models, African health leaders may unconsciously reproduce hospital-centric, acute-care paradigms inappropriate for managing chronic functional limitations (9, 11).

### Pillar 2 failure mechanism: workforce pipelines that start empty

The WHO strategy correctly identifies workforce shortages as critical (Sub-Saharan Africa: 0.04 physiotherapists per 10,000 population vs. global average 0.7) (4, 6). However, workforce development initiatives cannot succeed when medical graduates have never encountered rehabilitation as a career path or referral option.

Recent African initiatives demonstrate this challenge:

- *Ghana’s fellowship programs* showed promise but struggled with physician awareness and referral patterns (12)
- *Rwanda’s capacity building* required substantial investment in physician re-education alongside infrastructure (13)
- *South Africa’s relative success* (0.26 per 10,000) correlates directly with systematic curriculum integration (6, 14)

Medical students cannot choose career paths they’ve never encountered. The systematic exclusion of rehabilitation education becomes self-perpetuating, producing physicians who cannot appropriately refer patients to specialists they do not understand, working within health systems that cannot integrate services they do not value.

### Pillar 3 failure mechanism: services dismissed as inferior

Field evidence from Cameroon illuminates this mechanism starkly (15). Patients routinely travel over 1,000 kilometers—from Ngaoundéré requiring two nights of road travel, from Douala spanning 300 + kilometers—to consult rare rehabilitation specialists at modest public facilities. This “unique practitioner syndrome” reflects not individual practitioner excellence but profound system-level educational failures (15).

Patient testimonials reveal the consequences:

- *“This isn’t a hospital, it’s a man. He studied there [France]. It’s not the same”* (patient, 38 years, 7–9 hour journey)
- *“I came because they told me: ‘he’s the only one who made me walk again.’ You can’t not ry when you’ve tried everything”* (patient, 52 years, 5–7 h journey)

The pharmacotherapeutic default approach: Educational exclusion fosters dismissive attitudes toward rehabilitation that delegitimize evidence-based interventions. Physicians trained without rehabilitation exposure default to curative pharmaceutical solutions even when rehabilitation offers superior outcomes for stroke recovery, chronic pain management, and post-infectious sequelae—precisely the conditions driving Africa’s epidemiological transition (3, 6, 14).

The referral gap: Educational exclusion creates a complex barrier to appropriate care that extends beyond financial arrangements. This gap includes: (1) knowledge barriers where physicians fail to recognize conditions suitable for rehabilitation; (2) systemic barriers where referral pathways remain underdeveloped; (3) communication barriers between medical and rehabilitation professionals; and (4) problematic financial incentives, including physician-therapist commission arrangements (commonly 30–50% revenue sharing in Cameroon), where medical referrals become financial transactions rather than clinical decisions (15). This multifaceted breakdown of the referral system stems directly from physicians’ inability to evaluate rehabilitation competencies they never learned to recognize.

### Pillar 4 failure mechanism: invisible needs

Rehabilitation needs remain systematically undercounted when physicians cannot identify them (3, 11). The Global Burden of Disease 2019 study estimated that 2.4 billion people globally require rehabilitation services, with the highest unmet needs in low-income countries (3). However, these estimates rely on epidemiological modeling because surveillance systems fail to capture rehabilitation needs—a failure rooted in provider knowledge gaps.

#### Data systems reflect their designers’ knowledge

When health information architects lack rehabilitation training, they create surveillance systems that capture pharmaceutical prescriptions and surgical procedures but omit functional status assessments and rehabilitation referrals. The resulting data invisibility reinforces policy neglect.

### Pillar 5 failure mechanism: financing mechanisms for undervalued services

The WHO strategy urges member states to “identify financial mechanisms to integrate rehabilitation into essential health benefit packages” (2). However, healthcare financing mechanisms struggle to support services that referring physicians do not understand or value.

Health economics studies consistently demonstrate rehabilitation’s cost-effectiveness (3, 6). Yet African health ministries allocate minimal resources to rehabilitation—not because evidence is unavailable, but because decision-makers trained without rehabilitation exposure cannot comprehend its value proposition. You cannot finance what you cannot conceptualize.

## The economic paradox: low-cost solution to billion-dollar problem

Educational reform—defined here as the systematic integration of rehabilitation science and practice into medical curricula—offers exceptional return on investment. This reform encompasses four essential components:

1. Curriculum integration: Mandatory integration of 40–60 h of rehabilitation content into all medical schools’ core curricula, including theoretical frameworks (International Classification of Function), practical clinical exposure, and interprofessional collaborative practice.
2. Paradigm shift education: Deliberate emphasis on preventive, promotive, and rehabilitative approaches alongside the traditional curative model, challenging the existing pharmaceutical-surgical paradigm that dominates medical education.
3. Interprofessional education: Structured learning environments where medical students train alongside rehabilitation professionals, recognizing team-based competencies and appropriate referral pathways as core medical skills.
4. Systems-based practice: Integration of health systems strengthening content, teaching future physicians to understand rehabilitation’s role within comprehensive healthcare delivery, including community-based approaches and referral systems.

The marginal cost of implementing these educational reforms remains minimal—requiring primarily curriculum modification rather than extensive infrastructure development (14, 16), while transforming physicians’ fundamental understanding of complete healthcare delivery

In contrast:

- *Pillar 3 (service expansion)* requires construction of facilities, procurement of equipment, and sustained operational funding
- *Pillar 5 (resource mobilization)* requires years of advocacy and sustained financial commitments
- *Pillar 2 (workforce training)* requires establishing new training programs with specialized faculty

Curriculum integration amplifies all other investments. A physician trained to recognize rehabilitation needs becomes a force multiplier: appropriate referrals, interdisciplinary collaboration, evidence-based practice that extends rehabilitation’s reach throughout the health system (6, 16).

The University of Dschang experience provides preliminary evidence: when minimal 4-h rehabilitation exposure was introduced in 2021, student response was notable, with measurable increases in students expressing interest in rehabilitation careers (7). This transformation occurred through exposure alone, not elaborate recruitment campaigns.

## Successful African models: the educational common denominator

Comparative analysis of African success stories reveals educational reform as the consistent differentiating factor:

### South Africa: systematic integration

Deliberate curriculum integration across medical schools contributed to relatively robust rehabilitation services (0.26 physiotherapists per 10,000 inhabitants) (6, 14). The Health Professions Council of South Africa (HPCSA), the national regulatory body, mandates the inclusion of rehabilitation in all curricula training medical students, as specified in their official educational guidelines: “An approved educational institution shall create a curriculum which must achieve the following: preparing a student for health promotion, the prevention or treatment of illness and rehabilitation of impairment” (14). This regulatory requirement creates a physician workforce capable of appropriate referrals and system integration.”

### Rwanda: educational investment alongside infrastructure

Rwanda’s rehabilitation capacity building explicitly prioritized physician education alongside infrastructure development (13). The revised medical education curriculum includes rehabilitation sciences as core content, not elective exposure (13). This educational foundation enabled successful implementation of community-based rehabilitation models.

### Ghana: fellowship programs built on curriculum reform

Ghana’s postgraduate fellowship programs in Physical Medicine and Rehabilitation demonstrate how curriculum reform expands professional capacity (12, 16). However, these programs’ sustainability depends on undergraduate exposure creating sufficient interest to fill training pipelines.

### Morocco: measuring knowledge gaps to guide reform

Recent Moroccan research systematically documented knowledge gaps regarding Physical Medicine and Rehabilitation among training doctors and medical students (10). This evidence-based approach to curriculum reform, led by rehabilitation physicians like Dr. Abderrazak Hajjioui, provides a model for targeted educational interventions (10, 17).

### Senegal: emerging recognition

The HOGIP Medical Center in Dakar now offers comprehensive physical medicine and functional rehabilitation services (18), supported by curriculum integration at Université Cheikh Anta Diop that trains physicians to recognize rehabilitation as essential healthcare.

#### Common pattern

These successes share educational systems that train physicians to understand and value rehabilitation as essential healthcare, not luxury care for the privileged.

Table 1 summarizes the educational differentiators between successful and struggling African rehabilitation systems, demonstrating curriculum integration as the consistent predictor of implementation success.

**TABLE 1:** Comparative analysis: educational integration as predictor of rehabilitation system success in Africa.

Indicator	Successful systems	Struggling systems	Educational differentiator
Countries	South Africa, Rwanda, Ghana, Morocco, Senegal	Cameroon, most Central Africa	Curriculum integration
Workforce density	0.15–0.26 per 10,000	0.01–0.04 per 10,000	Training pipelines filled by interested students
Medical curriculum	Mandatory modules (20–60 h)	Absent or optional (0–4 h)	Core competency vs. elective
Physician knowledge	High (Morocco: structured assessment)	Low (Morocco study: 65% knowledge gaps)	Systematic vs. absent education
Referral patterns	Appropriate, evidence-based	Inappropriate or absent; commission-based (30–50%)	Clinical vs. financial decisions
Service distribution	Urban + rural networks	Urban-only; patients travel 300–1,000 km	Decentralized referral capacity
Implementation success	WHO pillars progressing	Pillar failures despite policy	Educational foundation present vs. absent
Cost-effectiveness recognition	Understood by providers	Dismissed; pharmaceutical default	Value comprehension vs. ignorance
Patient access	Improving systematically	“Unique practitioner syndrome”	System-level vs. individual practitioner
Policy integration	Rehabilitation in essential benefits	Excluded from priority services	Policymaker understanding vs. unfamiliarity
Data capture	Rehabilitation needs documented	Needs invisible in surveillance	Provider identification capacity
Infrastructure utilization	High (demand-driven expansion)	Low (underutilized facilities)	Educated referrers create demand
Educational investment timeline	Pre-2010 integration	Post-2020 minimal efforts	Sustained vs. nascent reform
Return on investment	High (amplifies all interventions)	Low (isolated interventions fail)	Educational multiplier effect

Successful systems consistently demonstrate medical curriculum integration predating rehabilitation system expansion, while struggling systems attempt service delivery and workforce development without educational foundation. This pattern persists across diverse economic contexts (Rwanda vs. South Africa), suggesting curriculum integration as necessary condition for sustainable implementation.

Table 1 compares five African countries demonstrating rehabilitation system success (South Africa, Rwanda, Ghana, Morocco, Senegal) with typical struggling systems (represented by Cameroon data but reflecting broader Central African patterns). Key metrics include: rehabilitation workforce density (therapists per 10,000 population), curriculum integration status (mandatory hours in medical school), physician knowledge levels (based on available assessments), referral patterns (appropriate vs. inappropriate), service accessibility (geographic distribution), and implementation outcomes. Data demonstrate consistent correlation between systematic curriculum integration and successful rehabilitation system implementation across diverse economic and infrastructure contexts. Sources: (6, 7, 10, 12–18).

## Contextually adapted tools undermined by knowledge gaps

The challenge extends beyond workforce numbers to workforce competencies. Several contextually adapted assessment and intervention tools have been developed for African settings—such as the Functional Assessment Scale for Elderly in Rural African Settings (EFAMRA) for geriatric evaluation (19), or culturally adapted neuropsychological batteries. These tools remain underutilized not because they lack validity, but because healthcare providers lack training to recognize their value or apply them appropriately.

This represents a tragic paradox: African researchers and clinicians develop contextually appropriate innovations that languish unused because the broader healthcare workforce cannot comprehend their utility. Educational exclusion thus undermines both service delivery and indigenous innovation.

## The digital opportunity: leapfrogging through technology

Africa’s mobile technology penetration (>70% in many regions) offers unprecedented opportunities for educational transformation (2, 4). Telemedicine platforms highlighted in the WHO strategy could simultaneously deliver education and clinical services, creating integrated learning-practice environments.

Proposed implementation model for digital education integration:

- Medical students observe real-time rehabilitation consultations through structured telemedicine platforms
- Rural practitioners access continuing education from urban expertise through guided modules and virtual mentoring
- Case-based learning with standardized patient scenarios addresses geographic distribution challenges
- Low-bandwidth platforms with offline capabilities enable knowledge transfer in resource-constrained settings without expensive infrastructure
- Integration of these digital approaches into formal medical curriculum with clear assessment metrics and faculty development

However, digital solutions cannot compensate for absent curricula. Technology amplifies existing knowledge; it cannot replace foundational education.

## The implementation timeline: why curriculum reform must come first

**FIGURE 1:**
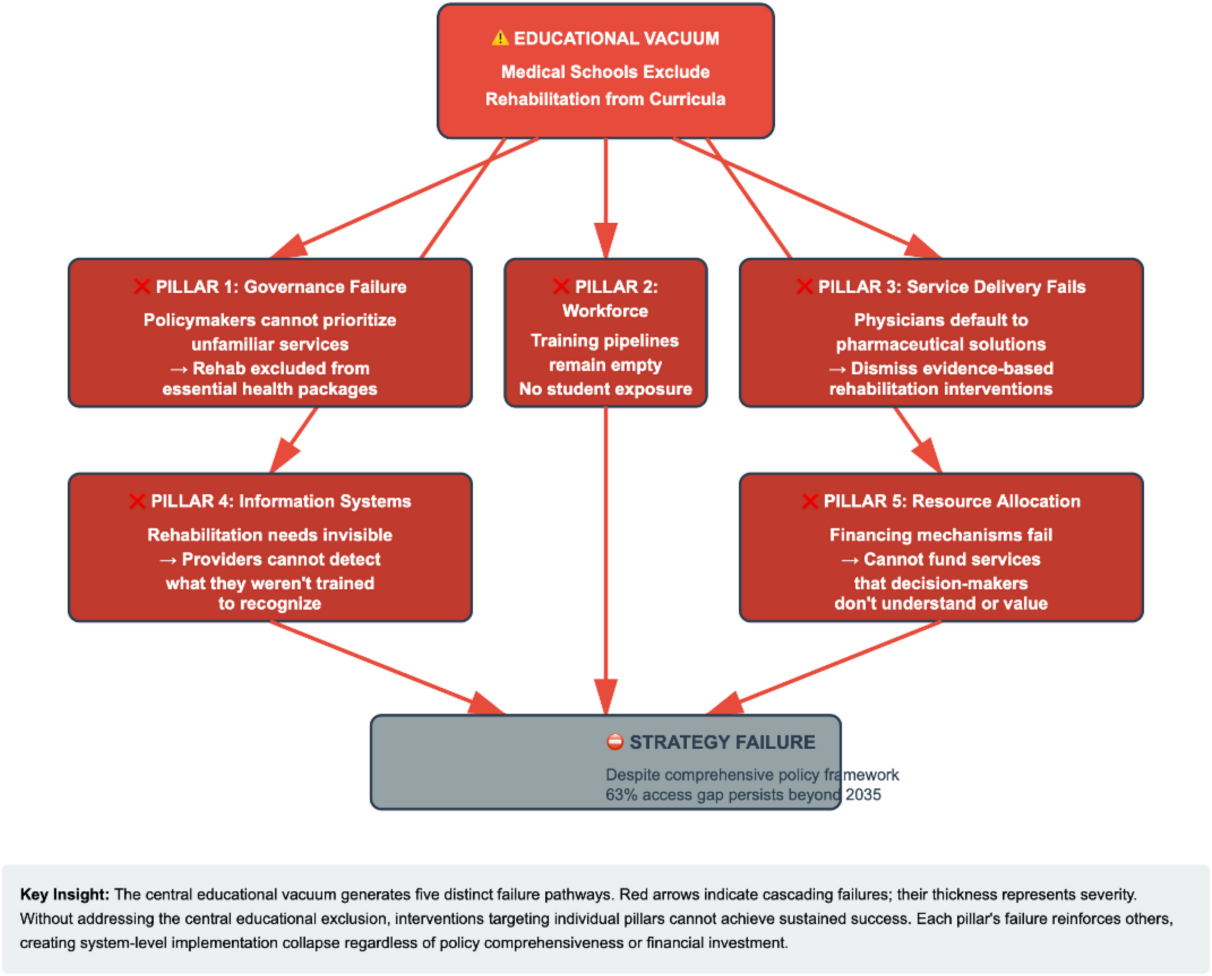
How educational exclusion sabotages all five WHO pillars. Cascading failure mechanisms: how educational exclusion undermines WHO strategic pillars. This diagram illustrates five distinct failure mechanisms through which systematic exclusion of rehabilitation from medical curricula undermines each WHO strategic pillar. The central “Educational Vacuum” (medical schools excluding rehabilitation) generates specific failure pathways for each pillar: (1) Governance fails when policymakers cannot prioritize unfamiliar services; (2) Workforce pipelines remain empty when students never encounter rehabilitation careers; (3) Service delivery defaults to pharmaceuticals when physicians dismiss rehabilitation as inferior; (4) Information systems render rehabilitation needs invisible when providers cannot identify them; (5) Resource allocation favors familiar interventions when decision-makers cannot conceptualize rehabilitation’s value. Red arrows indicate failure pathways; thickness represents severity of impact. Without addressing the central educational vacuum, interventions targeting individual pillars cannot achieve sustained success.

Figure 1 presents the proposed implementation framework showing educational reform as foundational infrastructure.

### Immediate actions (2025–2026): educational foundation

- *Mandate minimum rehabilitation modules* in all accredited medical programs (target: 40–60 curriculum hours)
- *Develop Regional Rehabilitation Education Consortium* coordinating curriculum resources across linguistic groups
- *Train medical school faculty* in rehabilitation medicine principles
- *Create standardized assessment tools* measuring medical student rehabilitation competencies

### Medium-term development (2027–2029): building on foundation

With educated physician workforce:

- *Pillar 1 initiatives succeed:* Policymakers prioritize rehabilitation appropriately
- *Pillar 2 initiatives succeed:* Training pipelines fill with interested candidates
- *Pillar 4 initiatives succeed:* Data systems capture rehabilitation needs accurately
- *Pillar 5 initiatives succeed:* Financing mechanisms support valued services

### Long-term transformation (2030–2035): sustained implementation

- *Pillar 3 initiatives succeed:* Service delivery expands sustainably
- Workforce capacity targets achieved
- Population health outcomes demonstrate measurable improvements
- 63% access gap progressively reduced

### Without foundational educational reform (2025–2026), subsequent pillars construct on unstable ground, risking structural failure regardless of resource investment

**FIGURE 2:**
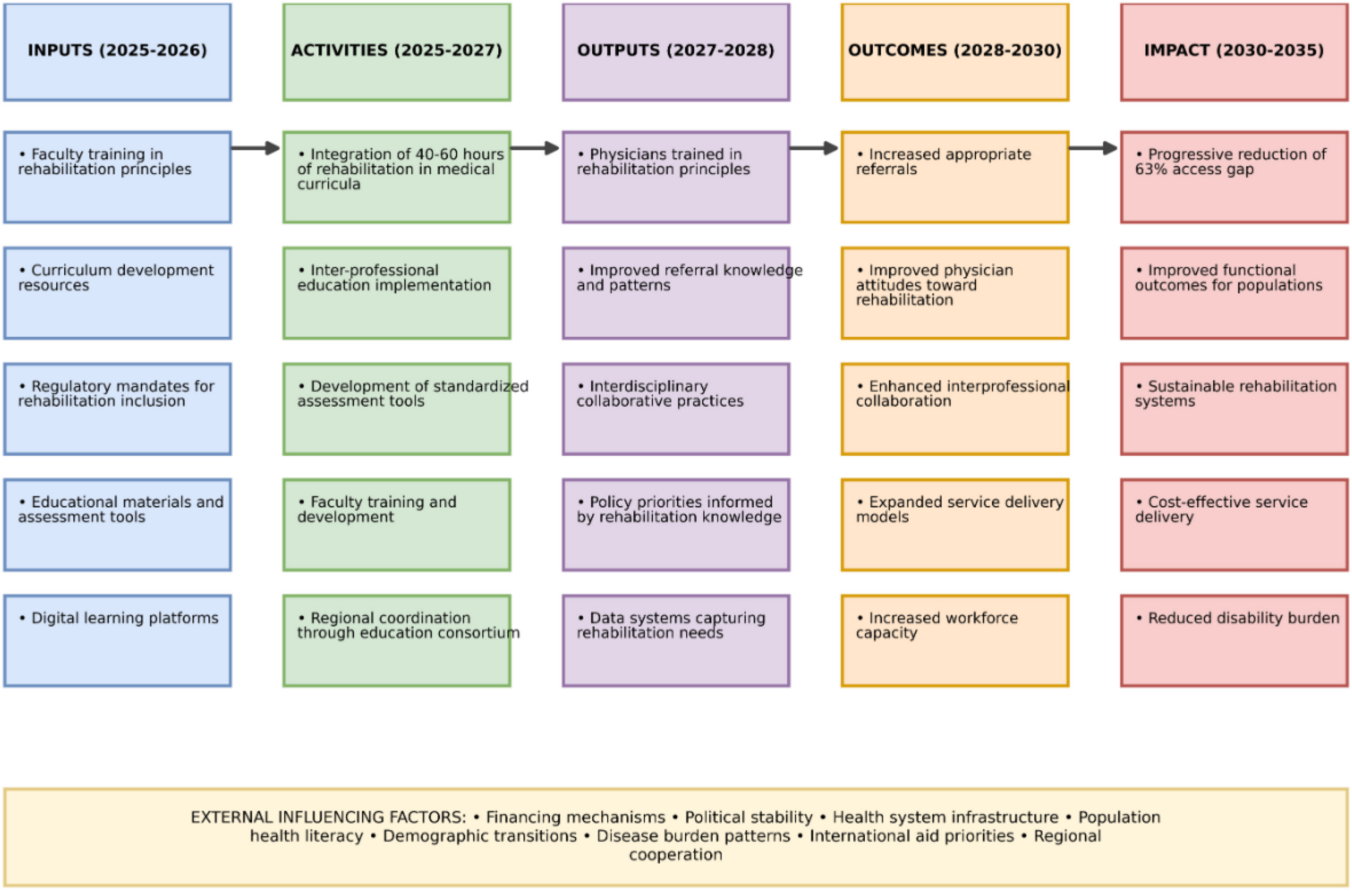
Logic model for educational reform as foundation for WHO AFRO strategy 2025–2035.

Figure 2 presents the proposed implementation framework as a logic model, showing how educational reform serves as foundational infrastructure supporting all five WHO strategic pillars. This logic model illustrates the causal pathway from inputs (curriculum reform resources) through activities (educational integration), outputs (trained physicians), outcomes (improved service delivery), to ultimate impact (reduced rehabilitation access gap).

This logic model illustrates the causal pathway through which medical education reform serves as the essential foundation for the successful implementation of the WHO AFRO Rehabilitation Strategy. The temporal progression from 2025 to 2035 is structured in five interdependent phases:

*INPUTS (2025–2026):* Fundamental resources required to initiate educational reform, representing minimal investment compared to infrastructural interventions while establishing the necessary foundation.

*ACTIVITIES (2025–2027):* Transformative actions that integrate rehabilitation as an essential component of medical curricula, promoting a paradigm shift from the curative model toward a more holistic approach.

*OUTPUTS (2027–2028):* Direct and measurable results of educational reform, creating the knowledge base needed to simultaneously support all five WHO strategic pillars.

*OUTCOMES (2028–2030):* Systemic changes resulting from a medical workforce sensitized to rehabilitation principles, transforming clinical and administrative practices across health systems.

*IMPACT (2030–2035):* Ultimate objectives of the strategy, demonstrating how early educational interventions progressively lead to substantial reduction of the 63% access gap.

The external influencing factors represent contextual variables that may facilitate or hinder progression through this model, requiring strategic adaptations according to national and regional contexts.

This logic model provides African decision-makers with a sequential and structured roadmap, prioritizing low-cost/high-impact interventions upstream to maximize return on investment and ensure sustainability across all five WHO strategic pillars.

## Regional coordination: opportunity for accelerated transformation

Rather than fragmented country-by-country reforms, the WHO strategy’s emphasis on regional coordination creates opportunities for educational transformation at scale (2, 4).

Proposed regional rehabilitation education consortium functions:

- Establish minimum curriculum standards applicable across diverse contexts
- Share educational resources across Francophone and Anglophone systems
- Create faculty exchange programs leveraging existing expertise (Morocco, South Africa, Rwanda)
- Develop digital learning platforms accessible across resource gradients
- Coordinate research documenting educational interventions’ effectiveness

*Institutional models exist:* The West African Health Organization (WAHO) and East, Central and Southern African Health Community (ECSA-HC) provide frameworks for coordinated educational initiatives. Rehabilitation curriculum integration could become regional standard within 18–24 months of coordinated effort.

## From conceptual framework to operational solution: a structured curriculum model

**FIGURE 3:**
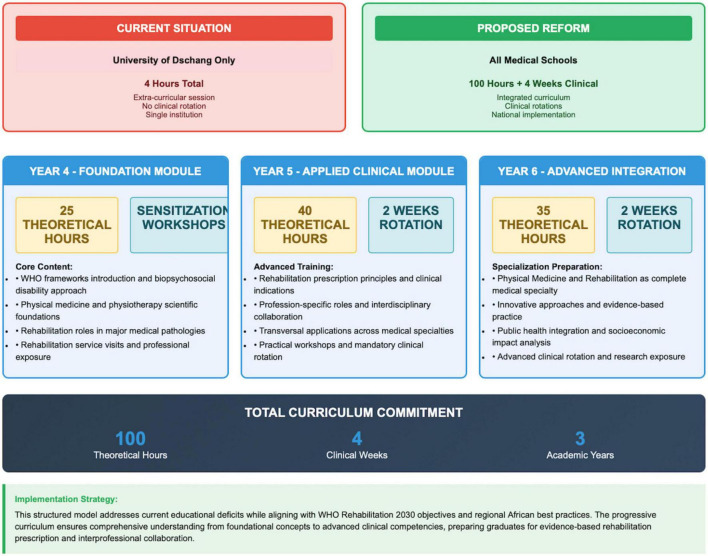
Current state vs. proposed reform model for Central African medical education.

While the preceding analysis establishes the critical need for educational reform and its timing relative to other interventions, a fundamental question remains unanswered: What precisely should this reformed curriculum contain? Drawing from successful models in South Africa, Rwanda, and Ghana, as well as contextual adaptation for Central African realities, Figure 3 presents an operational blueprint for transforming rehabilitation education within medical curricula.

This structured model advances beyond conceptual recommendations to provide a detailed, implementable pathway that addresses four essential components: theoretical foundations, clinical exposure, interprofessional collaboration, and progressive competency development. Rather than prescribing a one-size-fits-all approach, the proposed framework integrates rehabilitation across three academic years, ensuring systematic exposure that builds from core concepts to advanced integration. This progressive structure maintains implementability across diverse resource contexts while establishing minimum requirements to achieve physician competency in rehabilitation principles and referral patterns.

The contrast between current regional practice and the proposed model illustrates the magnitude of transformation required. Moving from isolated exposure at a single institution (University of Dschang’s exceptional yet insufficient 4 h) to a comprehensive 100-h curriculum with mandatory clinical rotations represents an ambitious yet achievable target. This model deliberately structures rehabilitation education across the medical curriculum to create sustained engagement rather than episodic exposure. The year-by-year progression from sensitization (year 4) to applied clinical practice (year 5) to advanced integration (year 6) allows for gradual incorporation into existing curricula while ensuring competency development meets WHO standards. Unlike resource-intensive interventions, this educational blueprint requires primarily faculty development and curriculum restructuring rather than extensive infrastructure investment, aligning with the low-cost, high-impact approach identified in Figure 2.

## Evidence-based implementation: African case studies supporting curriculum reform

The proposed curriculum model is not merely theoretical but draws directly from emerging African evidence demonstrating successful implementation of similar educational reforms with measurable impacts on rehabilitation service delivery. Several recent studies provide compelling evidence for the feasibility and effectiveness of systematic rehabilitation integration into medical education across diverse African contexts.

Moumeni’s research on emotional robotics in neurorehabilitation demonstrates how even limited technological resources can transform healthcare education when integrated into systematic curriculum frameworks (20). This study exemplifies how innovative approaches can bridge resource gaps through strategic educational reform, creating what the author terms “contextual innovation pathways” that maximize impact without requiring extensive infrastructure investment.

Particularly relevant to the proposed implementation timeline is Moumeni’s structured approach to acute-phase stroke rehabilitation (21, 22), which provides a detailed curriculum framework with day-by-day rehabilitation content adaptable to medical education. These extensively documented protocols offer ready-made educational modules that African medical schools could immediately incorporate into existing curricula, addressing the common barrier of content development resources. As the author notes: “Standardized rehabilitation protocols provide not just clinical guidance but educational infrastructure that accelerates curriculum integration across resource-diverse settings” (21).

The feasibility of cross-continental knowledge transfer is further validated by Moumeni’s comprehensive analysis of neuroplasticity principles adapted to African contexts (23). This research demonstrates how European biomedical principles can be effectively contextualized for African implementation (24) while maintaining evidence-based foundations—precisely the approach required for successful curriculum reform across Central Africa. The author’s emphasis on “translational educational pathways” provides a conceptual framework for the regional coordination proposed in Figure 3.

Most recently, Moumeni and colleagues’ innovative family-based rehabilitation approach (25) illustrates how educational reform creates cascading benefits throughout healthcare systems. By systematically training both medical professionals and family members in basic rehabilitation principles, this study documented significant improvements in functional outcomes while demonstrating efficient resource utilization—key priorities identified in the WHO AFRO strategy.

These African-led, contextually-validated studies collectively support the operational viability of the proposed curriculum model, demonstrating that educational reform represents not merely theoretical potential but proven effectiveness across diverse implementation contexts. As healthcare systems across the continent grapple with limited resources and competing priorities, this growing evidence base increasingly identifies curriculum reform as the highest-return investment for sustainable rehabilitation integration.

## Limitations and counterarguments addressed

### Limitation: generalizability from Cameroon observations

The ethnographic data derive primarily from Cameroon’s experience (15). However, systematic reviews confirm similar patterns across Sub-Saharan Africa (4, 6), and successful examples (South Africa, Rwanda, Ghana, Morocco) validate the educational reform hypothesis across diverse contexts.

### Counterargument: “Infrastructure, not education, is the primary barrier”

This argument conflates necessary and sufficient conditions. Infrastructure is necessary but not sufficient. Rwanda’s experience demonstrates that infrastructure without educated referrers remains underutilized (13). Conversely, South Africa shows that educated physicians create demand that drives infrastructure investment (6, 14).

### Counterargument: “Rehabilitation is expensive; Africa cannot afford it”

Cost-effectiveness studies consistently demonstrate rehabilitation’s superior value compared to pharmaceutical-only approaches for chronic conditions (3, 6). The perception of rehabilitation as expensive luxury reflects educational gaps, not economic reality. Community-based rehabilitation models prove highly cost-effective when appropriately implemented (2, 11).

### Limitation: proposed interventions require empirical testing

Agreed. However, the risk of inaction exceeds the risk of evidence-informed intervention. The WHO strategy’s 2035 timeline leaves no time for prolonged piloting. Regional implementation with rigorous monitoring offers ethical path forward.

## Conclusion: the choice between transformation and replication

The WHO AFRO 2025–2035 strategy provides comprehensive policy architecture for rehabilitation transformation. The five-pillar framework is technically sound, evidence-informed, and appropriately ambitious. Yet comprehensive frameworks have failed before.

The question confronting African health leaders is stark: Will this strategy succeed where previous initiatives failed, or will it replicate historical implementation failures despite superior policy design?

The answer depends critically on confronting the educational exclusion that systematically undermines rehabilitation integration. Without urgent curriculum reform:

- *Pillar 1* will struggle as policymakers trained without rehabilitation exposure cannot prioritize it effectively
- *Pillar 2* will fail as training pipelines remain empty due to absent undergraduate exposure
- *Pillar 3* will stagnate as physicians dismiss rehabilitation in favor of pharmaceutical defaults
- *Pillar 4* will produce incomplete data as providers cannot identify needs they never learned to recognize
- *Pillar 5* will misallocate resources to services that educated providers would recognize as inferior to rehabilitation approaches

Educational reform is not an enhancement to the WHO strategy; it is the foundational infrastructure upon which all five pillars must rest.

African health leaders face a choice:

- *Replicate historical failures* by implementing comprehensive frameworks atop inadequate educational foundations
- *Transform implementation potential* by prioritizing curriculum reform as essential infrastructure deserving immediate investment

The marginal cost approaches zero. The return on investment exceeds that of any other intervention. The timeline permits rapid deployment. The only barrier is recognizing education as healthcare infrastructure, not afterthought.

The WHO strategy provides the policy framework for transformation. Educational reform provides the human capacity to achieve it. Without both, the 63% access gap will persist beyond 2035, and another generation of Africans will suffer preventable functional limitations.

## Key questions

**What is already known?**

- WHO AFRO’s 2025–2035 strategy targets 63% rehabilitation access gap through five strategic pillars
- Sub-Saharan Africa faces critical rehabilitation workforce shortages (0.04 physiotherapists per 10,000 vs. global average 0.7)
- Previous rehabilitation policy initiatives have struggled with implementation despite strong frameworks

**What are the new findings?**

- Medical schools across Central Africa systematically exclude rehabilitation from curricula, threatening strategy implementation
- Educational gaps create cascading failures across all five WHO strategic pillars simultaneously
- Field evidence documents “unique practitioner syndrome” where patients travel 1,000 + km for rare rehabilitation expertise
- Successful African examples (South Africa, Rwanda, Ghana, Morocco) share common foundation: educational systems that train physicians to value rehabilitation

**What do the new findings imply?**

- Educational reform must be recognized as foundational infrastructure, not optional enhancement
- Without curriculum integration, comprehensive policy frameworks risk implementation failure regardless of financing or infrastructure investment
- Regional coordination offers opportunity for accelerated educational transformation through shared curriculum resources
- Medical education reform represents highest return-on-investment intervention for achieving WHO strategy targets

## Data Availability

The original contributions presented in this study are included in this article/supplementary material, further inquiries can be directed to the corresponding author.
